# Elaboration, étude structurale et analyse CHARDI et BVS d’une nouvelle variété β-Na_9_Cr(MoO_4_)_6_ de type alluaudite

**DOI:** 10.1107/S205698901600774X

**Published:** 2016-05-20

**Authors:** Manel Sonni, Riadh Marzouki, Mohamed Faouzi Zid, Amira Souilem

**Affiliations:** a Laboratoire de Matériaux et Cristallochimie, Faculté des Sciences de Tunis, Université de Tunis El Manar, 2092 Manar II Tunis, Tunisia

**Keywords:** crystal structure, alluaudite structure, framework, BVS, CHARDI

## Abstract

The title compound, nonasodium chromium(III) hexakis[molybdate(VI)], β-Na_9_CrMo_6_O_24_, was prepared by solid-state reactions. This alluaudite-type structure is constituted of infinite layers formed by links between *M*_2_O_10_ (*M* = C/Na) dimers and MoO_4_ tetra­hedra. The Na^+^ and Cr^3+^ cations are located in the same site with, respectively, 0.25 and 0.75 occupancies. The layers are connected to each other through MoO_4_ sharing corners, resulting an in open three-dimensional framework with hexa­gonal-form cavities occupied by Na^+^ cations. The proposed structural model is supported by charge-distribution (CHARDI) and bond-valence-sum (BVS) analysis. All atoms are on general positions except for one Mo, two Na (site symmetry 2) and another Na site (site symmetry -1). A comparison is made with the similar structures Na_4_Co(MoO_4_)_3_, Na_2_Ni(MoO_4_)_2_, Cu_1.35_Fe_3_(PO_4_)_3_ and NaAgFeMn_2_(PO_4_)_3_.

## Abstract 2

This alluaudite-type structure is constituted of infinite layers formed by links between *M*_2_O_10_ (*M* = Cr1/Na1) dimers and MoO_4_ tetra­hedra. The Na^+^ and Cr^3+^ cations are located in the same genel site with, respectively, 0.25 and 0.75 occupancies. The layers are related by sharing corners with MoO_4_ tetra­hedra, resulting an in open three-dimensional framework with hexa­gonal-form cavities occupied by Na^+^ cations.

## Contexte chimique   

La recherche de nouveaux matériaux pouvant être potentiellement des conducteurs ioniques ou bien des échangeurs d’ions, a conduit à s’intéresser aux composés à charpentes mixtes formées d’octa­èdres *M*O_6_ (*M* = métal de transition bi- ou trivalent) et de tétraèdres MoO_4_.

En effet, la jonction entre ces différents polyèdres conduit à des matériaux à charpentes ouvertes mixtes présentant de nombreuses propriétés physico-chimiques intéressantes qui sont en relation directe avec leurs structures cristallines, notamment: LiFe(MoO_4_)_2_ (van der Lee *et al.*, 2008 ▸), Na_2_Ni(MoO_4_)_2_ (Klevtsova *et al.*, 1991 ▸), CsFe(MoO_4_)_2_ (Baza­rov *et al.*, 2010 ▸), β-NaFe_2_(MoO_4_)_3_ (Muessig *et al.*, 2003 ▸), K_2_Co_2_(MoO_4_)_3_ (Engel *et al.*, 2009 ▸), K_0.13_Na_3.87_MgMo_3_O_12_ (Ennajeh *et al.*, 2015 ▸). Ce domaine est loin d’être entièrement exploré et fait l’objet des travaux présentant des intérêts fondamentaux qu’appliqués (Savina *et al.*, 2014 ▸; Namsaraeva *et al.*, 2011 ▸). C’est dans ce cadre que nous avons exploré les systèmes *A*–Cr–Mo–O (*A* = cation monovalent) dans lesquels nous avons précédemment caractérisé une seule phase intéressante: Na_9_Cr(MoO_4_)_6_ de symétrie hexa­gonale [la forme α-Na_9_Cr(MoO_4_)_6_; Dridi *et al.*, 2015 ▸]. Un nouveau composé de symétrie monoclinique a été synthétisé par réaction à l’état solide. Le mode de préparation, la détermination de la structure par diffraction des rayons-X sur monocristal et la validation des données, réalisée par les deux modèles CHARDI et BVS, seront présentés dans ce travail. La nouvelle variété β-Na_9_Cr(MoO_4_)_6_, bien qu’elle appartient à la famille des Alluaudites, présente une formule différente de celle généralement adoptée pour cette série de composés: *AA*′*MM*′*X*_3_O_12_ (*A*, *M* = métaux; *A* = mono, *M* = bi- ou trivalent et *X* = Mo, P, As).

## Description de la structure   

**Figure 1:**
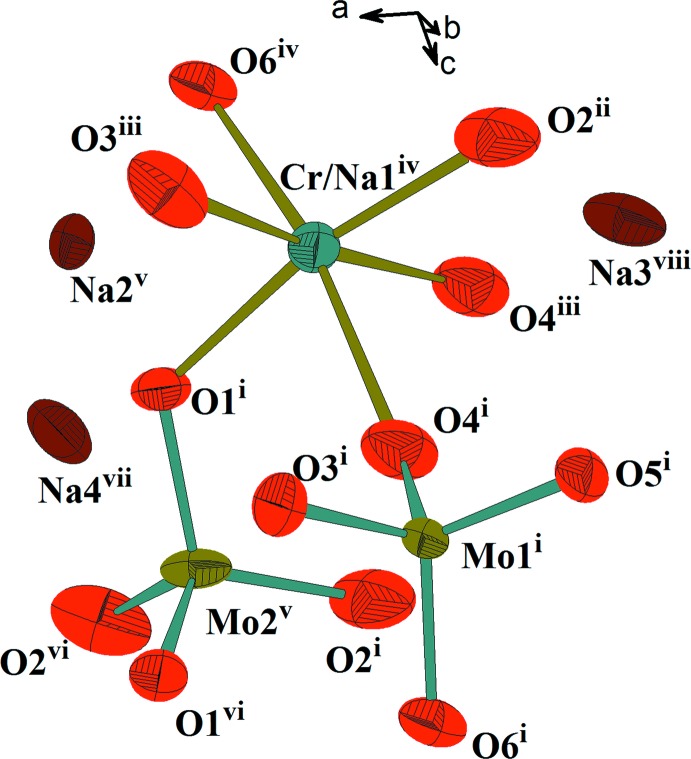
Unité structurale mettant en évidence les polyèdres de coordination dans l’unité asymétrique de β-Na_9_CrMo_6_O_24_. Les éllipsoïdes ont été définis avec 50% de probabilité. [Codes de symétrie: (i) −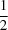 + *x*, 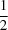 − *y*, 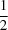 + *z*; (ii) 1 − *x*, *y*, 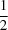 − *z*; (iii) −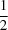 + *x*, −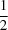 + *y*, *z*; (iv) −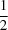 + *x*, −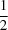 − *y*, −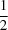 + *z*; (v) 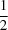 − *x*, 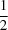 − *y*, 1 − *z*; (vi) 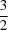 − *x*, 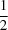 − *y*, 1 − *z*; (vii) 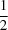 + *x*, −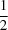 + *y*, *z*; (viii) 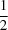 − *x*, −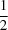 + *y*, 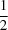 − *z*.]

**Figure 2:**
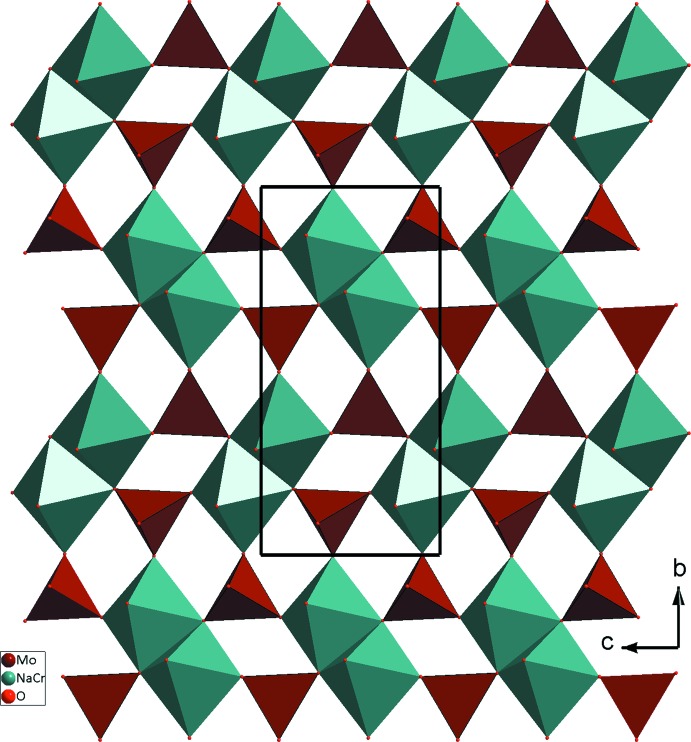
Projection d’une couche parallèlement au plan *bc*.

**Figure 3:**
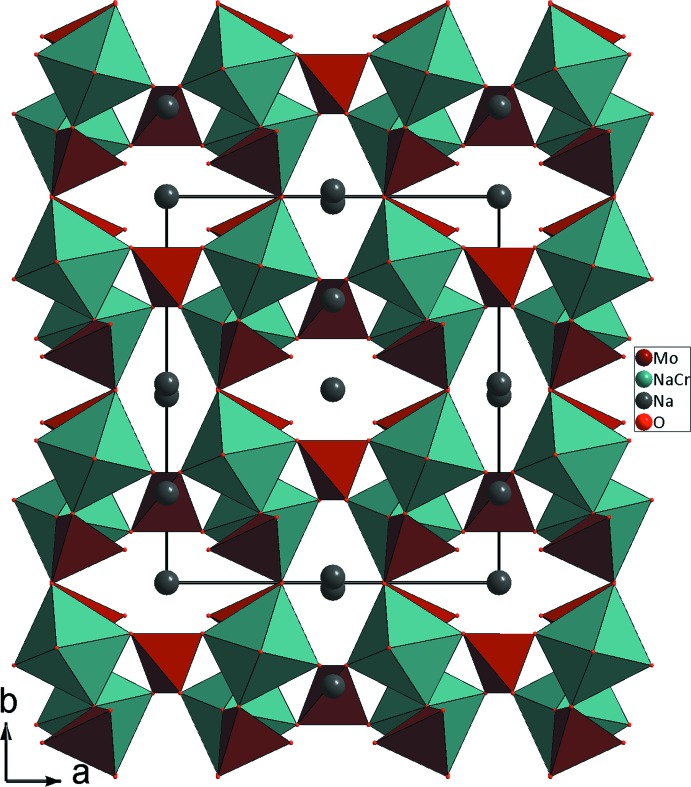
Projection de la structure de β-Na_9_CrMo_6_O_24_, selon *c*, mettant en évidence les cavités où résident les cation Na^+^.

L’unité structurale dans le composé β-Na_9_CrMo_6_O_24_ est constituée de deux tétraèdres MoO_4_ et d’un octa­èdre *M*O_6_ (*M* = Cr1/Na1) partageant des sommets oxygène. La compensation de charge est assurée par les cations Na^+^ (Fig. 1 ▸). La jonction des différents polyèdres, par ponts mixtes Mo—O—*M*1 (*M*1 = Cr1/Na1) et par arêtes entre octa­èdres, engendre des couches qui se lient à leur tour par insertion de tétraèdres Mo2O_4_ et conduit à une charpente tridimensionnelle. Chaque couche peut être décrite comme un assemblage de deux chaînes classiques de type *M*1Mo1O_8_ (*M*1 = Cr1/Na1). En effet, deux chaînes adjacentes se regroupent par mise en commun d’arêtes entre octa­èdres pour conduire à des chaînes doubles de type *M*1_2_Mo1_2_O_14_. Ces dernières se connectent moyennant des ponts mixtes de types Mo1—O—*M* pour donner des couches disposées parallèlement au plan *bc* (Fig. 2 ▸). Au sein d’une couche, les dimères appartenant à deux chaînes doubles adjacentes sont orientés perpendiculairement les uns par rapport aux autres. Cette disposition a favorisé l’orientation des tétraèdres Mo1O_4_, en *trans*, et a conduit à la jonction des couches par insertion des tétraèdres Mo2O_4_ entre elles par l’établissement des ponts mixtes Mo2—O—*M*_2_O_10_ (*M* = Cr1/Na1). Il en résulte une charpente tridimensionnelle laissant libre des cavités, à section hexa­gonale, où résident les cations alcalins Na^+^ (Fig. 3 ▸).

## Enquête de base de données   

**Figure 4:**
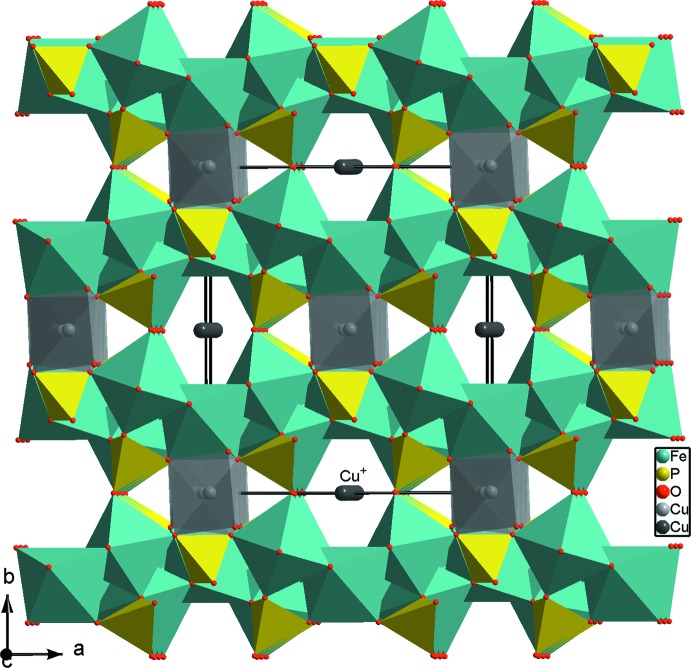
Projection de la structure de Cu_1,35_Fe_3_(PO_4_)_3_, selon *c*, montrant l’emplacement des ions Cu^2+^ et Cu^+^ dans le réseau.

**Figure 5:**
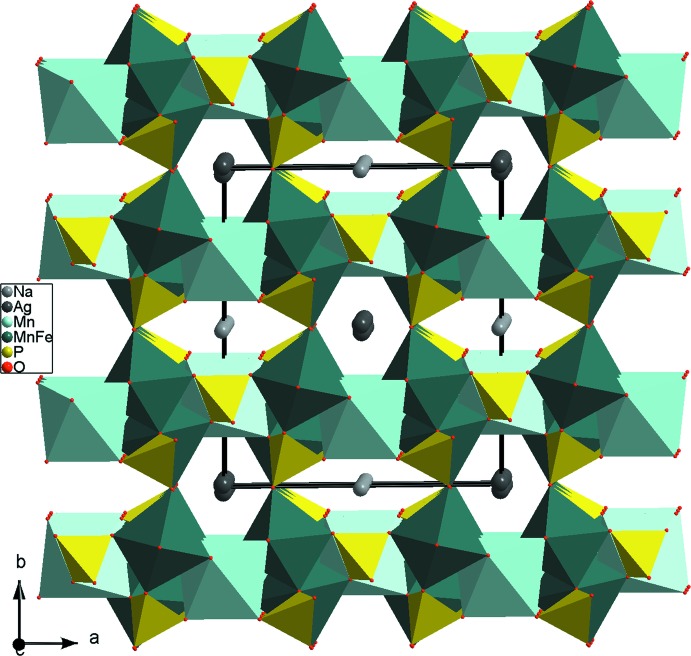
Projection de la structure de NaAgFeMn_2_(PO_4_)_3_, selon *c*, mettant en évidence la jonction des couches dans la charpente tridimensionnelle.

Cette variété, que nous avons appelé β-Na_9_CrMo_6_O_24_, est isoformulaire à celle précédemment obtenue α-Na_9_CrMo_6_O_24_ (Dridi *et al.*, 2015 ▸). Ces deux structures révèlent une différence nette. En effet, la forme bêta cristallise dans le système monoclinique, groupe d’espace *C*2/*c* et appartient à la famille des alluaudites possédant une charpentes tridimensionnelle alors que la forme alpha se caractérise par une structure à charpente unidimensionnelle et cristallise dans le système rhomboédrique, groupe d’espace *R* 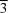 *c* avec les paramètres de maille suivants: *a* = 14,707 et *c* =19,175 Å. Un examen bibliographique des matériaux rencontrés dans la littérature et ayant une formulation générale de type *A*_*x*_ *MX*_3_O_12_ (*M* = Co, Ni, Mn, Fe et *X* = Mo, P), révèle une certaine similitude dans les structures de Na_4_Co(MoO_4_)_3_ (Nasri *et al.*, 2014 ▸), Na_2_Ni(MoO_4_)_2_ (Klevtsova *et al.*, 1991 ▸), Cu_1,35_Fe_3_(PO_4_)_3_ (Warner *et al.*, 1993 ▸) et NaAgFeMn_2_(PO_4_)_3_ (Daidouh *et al.*, 2002 ▸). Ces matériaux diffèrent essentiellement par l’occupation et la répartition des sites octa­édriques. En effet, dans la structure de Cu_1,35_Fe_3_(PO_4_)_3_ (Fig. 4 ▸) l’ion Cu^2+^ occupe le canal en remplaçant l’ion sodium Na3^+^ situé à l’origine de la maille et le cation Cu^+^ joue le rôle de l’ion alcalin Na4^+^ dans le composé étudié β-Na_9_Cr(MoO_4_)_6_. Alors que dans la structure de NaAgFeMn_2_(PO_4_)_3_, les ions Mn^2+^ forment des octa­èdres qui s’associent par ponts mixtes de type Mn—O—P ou bien Mn—O—*M* (*M* = Fe/Mn) et renforcent la jonction des couches dans la charpente tridimensionnelle (Fig. 5 ▸).

Si on se limite, à une sphère de coordination de rayon égal à 3 Å moyennant le programme *DIAMOND* (Brandenburg & Putz, 2001 ▸), on montre que les polyèdres Na2O_6_, Na3O_6_ et Na4O_4_ sont irréguliers (tableau 1 ▸), mais les moyennes des distances Na—O, dans la structure sont conformes à celles rencontrées dans la littérature. De plus, le calcul des différentes valences des liaisons utilisant la formule empirique de Brown (Brown & Altermatt, 1985 ▸) vérifie bien les valeurs de charges des ions: Mo1 (6,09), Mo2 (6,15), Cr1/Na1 (1,69), Na2 (1,12), Na3 (0,87), Na4 (0,76), attendues dans la phase étudiée.

Le modèle structural proposé, particulièrement la distribution au site *M*1(Cr1/Na1), est confirmé par les deux modèles de validation: la somme des valences de liaisons BVS (Brown, 2002 ▸; Adams, 2003 ▸) et la méthode de distribution de charges CHARDI (Nespolo *et al.*, 2001 ▸; Nespolo, 2001 ▸) (tableau 2 ▸). Les valeurs de charges calculées *Q*(*i*) et de valences *V*(*i*) sont en bon accord avec les degrés d’oxydation pondérés par les taux d’occupation. Le facteur de dispersion σ_cat_ (Nespolo, 2001 ▸) qui mesure la déviation des charges cationiques calculées est égal à 2,5%.

## Synthèse et cristallisation   

**Figure 6:**
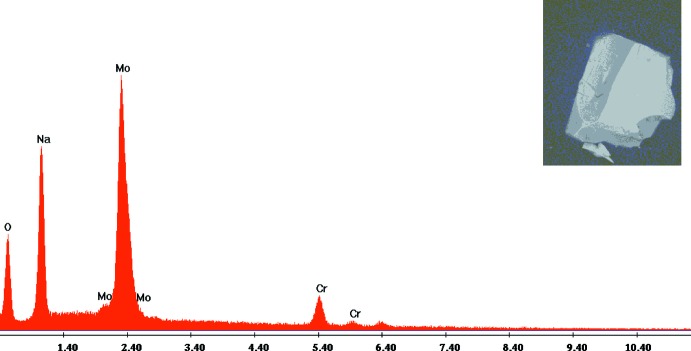
Analyse qualitative au MEB et morphologie d’un cristal de la variété β-Na_9_CrMo_6_O_24_.

Au cours de l’investigation des diagrammes *A*–Mo–Cr–O (*A* = Li, Na, Ag) un nouveau composé de fomulation β-Na_9_CrMo_6_O_24_ a été élaboré. Les cristaux ont été obtenus par voie sèche en broyant dans un mortier en agate les réactifs NaNO_3_, Cr(NO_3_)_3_·9H_2_O et (NH_4_)_6_Mo_7_O_24_·4H_2_O dans les rapports molaires Na:Cr:Mo égalent à 6:1:4, respectivement. Le mélange obtenu a subit une calcination à 673 K afin d’éliminer les produits volatils notamment: NO_2_, NH_3_ et H_2_O. Ensuite, la poudre résiduelle a subit un broyage fin puis remis dans le four à une température proche de la fusion à 973 K pendant trois jours pour favoriser la germination et la croissance des cristaux. Après refroidissement du four, des cristaux de forme parallélépipédique et de taille optimale pour la collecte des données, ont été obtenus. Une analyse qualitative au MEB de marque FEI et de type Quanta 200 confirme la présence des différents éléments chimiques attendus: Mo, Cr, Na et l’oxygène (Fig. 6 ▸).

## Affinement   

Un cristal sélectionné sous microscope polarisant, de bonne qualité, a servi pour les mesures des intensités (tableau 3 ▸). Un examen des facteurs géométriques montre que la distance *M*1—O égale à 2,208 Å (*M*1 = Cr1/Na1) s’avère une moyenne entre celles Cr1—O et Na1—O rencontrées dans la littérature. Un affinement des contraintes EADP et EXYZ ainsi que SUMP autorisées par le programme *SHELXL97* (Sheldrick, 2008 ▸), a conduit vers des taux d’occupation du Cr1 et Na1 égaux à 0,244 (6) et 0,755 (7) respectivement. Les conditions de la neutralité électrique pour le matériau nous ont encouragé à fixer ces derniers à 25% pour le Cr1 et 75% pour le Na1. A la fin de la résolution, les densités d’électrons maximum et minimum restants dans la Fourier-différence sont acceptables et sont situées respectivement à 0,78 Å de Mo1 et 0,89 Å de Mo2. De plus, l’affinement final conduit à des ellipsoïdes bien définis.

## Supplementary Material

Crystal structure: contains datablock(s) I. DOI: 10.1107/S205698901600774X/ru2067sup1.cif

Structure factors: contains datablock(s) I. DOI: 10.1107/S205698901600774X/ru2067Isup2.hkl

CCDC reference: 1479074

Additional supporting information:  crystallographic information; 3D view; checkCIF report

## Figures and Tables

**Table 1:** Longueurs de liaison sélectionnés (Å)

Mo1—O5	1.736 (4)	Cr1—O3^v^	2.240 (5)
Mo1—O6	1.742 (4)	Cr1—O4^iii^	2.335 (5)
Mo1—O3	1.762 (4)	Na2—O1^vi^	2.375 (3)
Mo1—O4	1.762 (4)	Na2—O6^vii^	2.401 (4)
Mo2—O1^i^	1.750 (3)	Na2—O5^viii^	2.472 (4)
Mo2—O1^ii^	1.750 (3)	Na3—O5	2.494 (3)
Mo2—O2^ii^	1.756 (5)	Na3—O2^ix^	2.532 (4)
Cr1—O2^iii^	2.132 (6)	Na3—O5^i^	2.683 (4)
Cr1—O1^iv^	2.153 (4)	Na4—O3^ii^	2.513 (5)
Cr1—O6	2.181 (4)	Na4—O3^x^	2.575 (5)
Cr1—O4^iv^	2.210 (4)		

Symmetry codes: (i) 
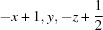
; (ii) 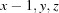; (iii) 
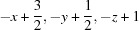
; (iv) 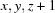; (v) 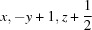; (vi) 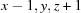; (vii) 
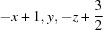
; (viii) 
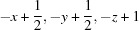
; (ix) 
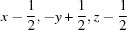
; (x) 
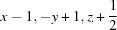
.

**Table 2:** Analyse CHARDI et BVS des cations dans le composé β-Na_9_CrMo_6_O_24_

Cation	*q*(*i*)·sof(*i*)	*Q*(*i*)	*V*(*i*)	CN(*i*)	ECoN(*i*)	*d*_moy_	*d*_med_
Mo1	6,000	6,130	6,0988	4	3,99	1,7502	1,7507
Mo2	6,000	6,150	6,1532	4	4,00	1,7525	1,7524
*M*(Cr/Na)	1,50	1,300	1,6875	6	5,82	2,2085	2,2084
Na2	1,000	1,010	1,1166	6	5,94	2,4158	2,4159
Na3	1,000	1,010	0,8719	6	6,14	2,5699	2,5695
Na4	1,000	0,970	0,7578	4	5,02	2,5441	2,5443

*q*(*i*) = nombre d’oxydation formel; sof(*i*) = taux d’occupation du site; CN(*i*) = nombre de coordination classique; *Q*(*i*)=charge calculée; *V*(*i*)= valence calculée; ECoN(*i*) = nombre de coordination effectif; *d*_moy_(*i*) = distance arithmétique moyenne; *d*_med_(*i*) = distance pondérée moyenne; σ_cat_ = facteur de dispersion sur les charges cationiques; σ_cat_ = [Σ_i_(*q*_i_ − *Q*_i_)^2^/*N* − 1]^1/2^ = 0,025.

**Table 3:** Détails expérimentaux

Données crystallines	
Formule chimique	Na_9_CrMo_6_O_24_
*M*_r_	1218,55
Système cristallin, groupe d’espace	Monoclinique, *C*2/*c*
Température (K)	298
*a*, *b*, *c* (Å)	12,655 (2), 13,578 (2), 7,1405 (8)
β (°)	112,58 (2)
*V* (Å^3^)	1132,9 (3)
*Z*	2
Type de rayonnement	Mo *K*α
μ (mm^−1^)	3,96
Taille des cristaux (mm)	0,28 × 0,22 × 0,14

Collection de données	
Diffractomètre	Enraf–Nonius CAD-4
Correction d’absorption	ψ scan (North *et al.*, 1968 ▸)
*T*_min_, *T*_max_	0,356, 0,574
Nombre de réflexions mesurées, indépendantes et observées [*I* > 2σ(*I*)]	2518, 1231, 1065
*R*_int_	0,023
(sin θ/λ)_max_ (Å^−1^)	0,638

Affinement	
*R*[*F* ^2^ > 2σ(*F* ^2^)], *wR*(*F* ^2^), *S*	0,028, 0,080, 1,06
Nombre de réflexions	1231
Nombre de paramètres	97
Δρ_max_, Δρ_min_ (e Å^−3^)	0,92, −0,98

Programmes informatiques: *CAD-4 EXPRESS* (Duisenberg, 1992 ▸; Macíček & Yordanov, 1992 ▸), *XCAD4* (Harms & Wocadlo, 1995 ▸), *SHELXS97* et *SHELXL97* (Sheldrick, 2008 ▸), *DIAMOND* (Brandenburg & Putz, 2001 ▸), *WinGX* (Farrugia, 2012 ▸) et *publCIF* (Westrip, 2010 ▸).

## supplementary crystallographic information

### Crystal data

**Table:**

Na_9_CrMo_6_O_24_	*F*(000) = 1134
*M**_r_* = 1218.55	*D*_x_ = 3.572 Mg m^−^^3^
Monoclinic, *C*2/*c*	Mo *K*α radiation, λ = 0.71073 Å
Hall symbol: -C 2yc	Cell parameters from 25 reflections
*a* = 12.655 (2) Å	θ = 10–15°
*b* = 13.578 (2) Å	µ = 3.96 mm^−^^1^
*c* = 7.1405 (8) Å	*T* = 298 K
β = 112.58 (2)°	Prism, red
*V* = 1132.9 (3) Å^3^	0.28 × 0.22 × 0.14 mm
*Z* = 2	

### Data collection

**Table:**

Enraf–Nonius CAD-4 diffractometer	1065 reflections with *I* > 2σ(*I*)
Radiation source: fine-focus sealed tube	*R*_int_ = 0.023
Graphite monochromator	θ_max_ = 27.0°, θ_min_ = 2.3°
ω/2θ scans	*h* = −16→12
Absorption correction: ψ scan (North *et al.*, 1968)	*k* = −1→17
*T*_min_ = 0.356, *T*_max_ = 0.574	*l* = −9→9
2518 measured reflections	2 standard reflections every 120 min
1231 independent reflections	intensity decay: 1.3%

### Refinement

**Table:**

Refinement on *F*^2^	Primary atom site location: structure-invariant direct methods
Least-squares matrix: full	Secondary atom site location: difference Fourier map
*R*[*F*^2^ > 2σ(*F*^2^)] = 0.028	*w* = 1/[σ^2^(*F*_o_^2^) + (0.0433*P*)^2^ + 5.0898*P*] where *P* = (*F*_o_^2^ + 2*F*_c_^2^)/3
*wR*(*F*^2^) = 0.080	(Δ/σ)_max_ < 0.001
*S* = 1.06	Δρ_max_ = 0.92 e Å^−^^3^
1231 reflections	Δρ_min_ = −0.98 e Å^−^^3^
97 parameters	Extinction correction: *SHELXL97* (Sheldrick, 2008), Fc^*^=kFc[1+0.001xFc^2^λ^3^/sin(2θ)]^-1/4^
0 restraints	Extinction coefficient: 0.0012 (2)

### Special details

**Table:**

Geometry. All esds (except the esd in the dihedral angle between two l.s. planes) are estimated using the full covariance matrix. The cell esds are taken into account individually in the estimation of esds in distances, angles and torsion angles; correlations between esds in cell parameters are only used when they are defined by crystal symmetry. An approximate (isotropic) treatment of cell esds is used for estimating esds involving l.s. planes.
Refinement. Refinement of F^2^ against ALL reflections. The weighted R-factor wR and goodness of fit S are based on F^2^, conventional R-factors R are based on F, with F set to zero for negative F^2^. The threshold expression of F^2^ > 2sigma(F^2^) is used only for calculating R-factors(gt) etc. and is not relevant to the choice of reflections for refinement. R-factors based on F^2^ are statistically about twice as large as those based on F, and R- factors based on ALL data will be even larger.

### Fractional atomic coordinates and isotropic or equivalent isotropic displacement parameters (Å^2^)

**Table:**

	*x*	*y*	*z*	*U*_iso_*/*U*_eq_	Occ. (<1)
Mo1	0.77009 (3)	0.39149 (3)	0.37667 (6)	0.02349 (17)	
Mo2	0.0000	0.21336 (4)	0.2500	0.0280 (2)	
Cr1	0.78758 (12)	0.34023 (11)	0.8756 (2)	0.0267 (3)	0.25
Na1	0.78758 (12)	0.34023 (11)	0.8756 (2)	0.0267 (3)	0.75
Na2	0.0000	0.2313 (2)	0.7500	0.0244 (5)	
Na3	0.5000	0.5000	0.0000	0.0409 (8)	
Na4	0.0000	0.5144 (4)	0.7500	0.0591 (12)	
O1	0.9590 (3)	0.2858 (3)	0.0309 (5)	0.0284 (7)	
O2	0.8920 (5)	0.1320 (3)	0.2490 (7)	0.0557 (13)	
O3	0.8458 (4)	0.5028 (3)	0.4008 (7)	0.0494 (12)	
O4	0.7828 (4)	0.3206 (4)	0.1796 (6)	0.0471 (11)	
O5	0.6258 (3)	0.4147 (3)	0.3165 (6)	0.0302 (8)	
O6	0.8308 (3)	0.3307 (3)	0.6084 (5)	0.0340 (8)	

### Atomic displacement parameters (Å^2^)

**Table:**

	*U*^11^	*U*^22^	*U*^33^	*U*^12^	*U*^13^	*U*^23^
Mo1	0.0186 (2)	0.0331 (3)	0.0158 (2)	−0.00152 (16)	0.00336 (16)	0.00283 (14)
Mo2	0.0463 (4)	0.0182 (3)	0.0140 (3)	0.000	0.0054 (2)	0.000
Cr1	0.0274 (8)	0.0318 (8)	0.0209 (7)	−0.0042 (6)	0.0092 (6)	−0.0020 (5)
Na1	0.0274 (8)	0.0318 (8)	0.0209 (7)	−0.0042 (6)	0.0092 (6)	−0.0020 (5)
Na2	0.0216 (13)	0.0264 (13)	0.0291 (14)	0.000	0.0142 (11)	0.000
Na3	0.052 (2)	0.0244 (14)	0.0282 (15)	0.0021 (13)	−0.0041 (14)	−0.0012 (12)
Na4	0.0232 (17)	0.120 (4)	0.0291 (17)	0.000	0.0045 (13)	0.000
O1	0.0319 (18)	0.0332 (19)	0.0195 (16)	0.0033 (15)	0.0092 (14)	0.0057 (14)
O2	0.073 (3)	0.035 (2)	0.042 (3)	−0.014 (2)	0.002 (2)	0.0113 (19)
O3	0.038 (2)	0.056 (3)	0.047 (3)	−0.018 (2)	0.009 (2)	0.015 (2)
O4	0.042 (2)	0.071 (3)	0.028 (2)	0.012 (2)	0.0122 (18)	−0.001 (2)
O5	0.0226 (18)	0.0288 (18)	0.035 (2)	0.0034 (14)	0.0067 (15)	0.0084 (15)
O6	0.032 (2)	0.039 (2)	0.0249 (19)	0.0100 (16)	0.0037 (15)	0.0038 (15)

### Geometric parameters (Å, º)

**Table:**

Mo1—O5	1.736 (4)	Na2—O1^i^	2.375 (4)
Mo1—O6	1.742 (4)	Na2—O6^vii^	2.401 (4)
Mo1—O3	1.762 (4)	Na2—O6^ii^	2.401 (4)
Mo1—O4	1.762 (4)	Na2—O5^viii^	2.472 (4)
Mo2—O1^i^	1.750 (3)	Na2—O5^ix^	2.472 (4)
Mo2—O1^ii^	1.750 (3)	Na3—O5	2.494 (3)
Mo2—O2^ii^	1.756 (5)	Na3—O5^x^	2.494 (3)
Mo2—O2^i^	1.756 (5)	Na3—O2^xi^	2.532 (4)
Cr1—O2^iii^	2.132 (6)	Na3—O2^xii^	2.532 (4)
Cr1—O1^iv^	2.153 (4)	Na3—O5^i^	2.683 (4)
Cr1—O6	2.181 (4)	Na3—O5^xiii^	2.683 (4)
Cr1—O4^iv^	2.210 (4)	Na4—O3^ii^	2.513 (5)
Cr1—O3^v^	2.240 (5)	Na4—O3^vii^	2.513 (5)
Cr1—O4^iii^	2.335 (5)	Na4—O3^xiv^	2.575 (5)
Na2—O1^vi^	2.375 (3)	Na4—O3^xv^	2.575 (5)
			
O5—Mo1—O6	111.06 (18)	O1^iv^—Cr1—O6	83.61 (14)
O5—Mo1—O3	110.5 (2)	O2^iii^—Cr1—O4^iv^	90.27 (19)
O6—Mo1—O3	106.8 (2)	O1^iv^—Cr1—O4^iv^	81.65 (16)
O5—Mo1—O4	108.13 (19)	O6—Cr1—O4^iv^	164.22 (18)
O6—Mo1—O4	111.0 (2)	O2^iii^—Cr1—O3^v^	97.49 (17)
O3—Mo1—O4	109.4 (2)	O1^iv^—Cr1—O3^v^	92.85 (16)
O1^i^—Mo2—O1^ii^	111.6 (2)	O6—Cr1—O3^v^	86.64 (17)
O1^i^—Mo2—O2^ii^	108.7 (2)	O4^iv^—Cr1—O3^v^	99.67 (19)
O1^ii^—Mo2—O2^ii^	112.77 (19)	O2^iii^—Cr1—O4^iii^	79.54 (17)
O1^i^—Mo2—O2^i^	112.77 (19)	O1^iv^—Cr1—O4^iii^	90.56 (15)
O1^ii^—Mo2—O2^i^	108.7 (2)	O6—Cr1—O4^iii^	90.24 (15)
O2^ii^—Mo2—O2^i^	102.0 (4)	O4^iv^—Cr1—O4^iii^	84.30 (17)
O2^iii^—Cr1—O1^iv^	167.83 (16)	O3^v^—Cr1—O4^iii^	175.10 (17)
O2^iii^—Cr1—O6	103.32 (18)		

Symmetry codes: (i) −*x*+1, *y*, −*z*+1/2; (ii) *x*−1, *y*, *z*; (iii) −*x*+3/2, −*y*+1/2, −*z*+1; (iv) *x*, *y*, *z*+1; (v) *x*, −*y*+1, *z*+1/2; (vi) *x*−1, *y*, *z*+1; (vii) −*x*+1, *y*, −*z*+3/2; (viii) −*x*+1/2, −*y*+1/2, −*z*+1; (ix) *x*−1/2, −*y*+1/2, *z*+1/2; (x) −*x*+1, −*y*+1, −*z*; (xi) *x*−1/2, −*y*+1/2, *z*−1/2; (xii) −*x*+3/2, *y*+1/2, −*z*+1/2; (xiii) *x*, −*y*+1, *z*−1/2; (xiv) *x*−1, −*y*+1, *z*+1/2; (xv) −*x*+1, −*y*+1, −*z*+1.
